# Cytogenetic analysis of three species of *Pseudacteon* (Diptera, Phoridae) parasitoids of the fire ants using standard and molecular techniques

**DOI:** 10.1590/S1415-47572009005000073

**Published:** 2009-12-01

**Authors:** Mónica G. Chirino, Patricia J. Folgarait, Lawrence E. Gilbert, Silvia Lanzavecchia, Alba G. Papeschi

**Affiliations:** 1 Centro de Estudios e Investigaciones, Universidad Nacional de Quilmes, Bernal, Buenos AiresArgentina; 2 Brackenridge Field Laboratory, University of Texas, AustinUSA; 3 Instituto de Genética, INTA, Castelar, Buenos AiresArgentina; 4 Laboratorio de Citogenética y Evolución, Facultad de Ciencias Exactas y Naturales, Universidad de Buenos Aires, Buenos AiresArgentina

**Keywords:** *Pseudacteon*, karyotype evolution, C-banding, fluorescent banding, FISH

## Abstract

*Pseudacteon* flies, parasitoids of worker ants, are being intensively studied as potentially effective agents in the biological control of the invasive pest fire ant genus *Solenopsis* (Hymenoptera: Formicidae). This is the first attempt to describe the karyotype of *P. curvatus* Borgmeier, *P. nocens* Borgmeier and *P. tricuspis* Borgmeier. The three species possess 2n = 6; chromosomes I and II were metacentric in the three species, but chromosome pair III was subtelocentric in *P. curvatus* and *P. tricuspis*, and telocentric in *P. nocens.* All three species possess a C positive band in chromosome II, lack C positive heterochromatin on chromosome I, and are mostly differentiated with respect to chromosome III. *P. curvatus* and *P. tricuspis* possess a C positive band, but at different locations, whereas this band is absent in *P. nocens.* Heterochromatic bands are neither AT nor GC rich as revealed by fluorescent banding. *In situ* hybridization with an 18S rDNA probe revealed a signal on chromosome II in a similar location to the C positive band in the three species. The apparent lack of morphologically distinct sex chromosomes is consistent with proposals of environmental sex determination in the genus. Small differences detected in chromosome length and morphology suggests that chromosomes have been highly conserved during the evolutionary radiation of *Pseudacteon.* Possible mechanisms of karyotype evolution in the three species are suggested.

## Introduction

The Phoridae, one of the most diverse families within the order Diptera, includes more than 26,000 min species, varying in life style from scavengers and predators to parasitoids (Disney, 1994; Gilbert and Jervis, 1998). The phorid genus *Pseudacteon* Coquillet are parasitoids, most of the species parasitizing *Solenopsis* Westwood fire-ants (Hymenoptera: Formicidae) (Disney, 1994). *Pseudacteon* flies are solitary parasitoids (Disney, 1994). The females inject a single egg into each worker ant, the third larval instar of which migrating into and later pupating within the host's cephalic capsule (Porter *et al.*, 1995; Porter, 1998).

*Solenopsis* has a cosmopolitan distribution (Disney, 1994; Patrock *et al.*, 2009). In South America, 22 *Pseudacteon* species have been reported as parasitizing fire-ants of the *Solenopsissaevissima* complex (Porter and Pesquero, 2001; Brown *et al.*, 2003; Folgarait *et al.*, 2005b; Calcaterra, 2007; Kronforst *et al.*, 2007). Currently, these flies are used as biological control agents against *Solenopsis invicta* Buren and *S*. *richteri* Forel fire-ants, native to Argentina (Mescher *et al.*, 2003; Ross *et al.*, 2008) and exotic pests in the United States (Porter *et al.*, 2004; Thead *et al.*, 2005; Morrison and Porter, 2006).

Despite numerous studies on the life-history traits of the genus *Pseudacteon*, including apparent environmental sex-determination in some species, cytogenetic studies are lacking. Dipteran species generally possess low diploid chromosome numbers, these ranging from 2n = 4 to 20, with modal numbers at 6 and 8 (due to several families of “lower” Diptera *i.e.* SO Nematocera) and 12 (with many species of the SO Brachycera and belonging to calyptrate and acalyptrate families) (White, 1973). Almost all species of Sarcophagidae and Calliphoridae (SO Brachycera, superfamily Oestroidea) have 2n = 12 with a pair of sex chromosomes XY (Parise-Maltempi and Avancini, 2000; 2001). In the family Drosophilidae as a whole (SO Brachycera, superfamily Acalyptrata) the primitive chromosome number appears to be 2n = 12 with five pairs of long acrocentric chromosomes (including the sex chromosome pair XY) and a small dot-like pair. Reduction in chromosome number has occurred many times independently by fusion of these elements (White, 1973). Many derived sex chromosome systems have been described and the evolutionary mechanisms of sex chromosome differentiation has been analyzed in *Drosophila* species (Steinemann and Steinemann, 2005). In Muscidae (SO Brachycera, superfamily Muscoidea) most species also possess 2n = 12 with XY sex chromosomes in the male. However, muscid species have been described with 2n = 10 chromosomes and lacking differentiated sex chromosomes (Parise-Maltempi and Avancini, 2001). In Phoridae (SO Brachycera, superfamily Phoroidea), *Megaselia scalaris* Loew and *M. spiracularis* Schmitz, the only 2 species that have been studied cytogenetically, possess the diploid number 2n = 6, with 2 metacentric and 1 telocentric chromosome pairs. They do not possess heteromorphic sex chromosomes and sex is determined by a single *Maleness* factor that can be located in any of the three linkage groups (Mainx, 1964; Traut and Willhoeft, 1990).

In order to evaluate whether the well-marked differences among *P. curvatus* Borgmeier*, P. nocens* Borgmeier and *P. tricuspis* Borgmeier in size, morphology, behavior and phylogenetic relations are also reflected in their karyotypes, our aim was to describe the karyotype of these species by using standard and molecular cytogenetic techniques. Descriptive values of the karyotype, heterochromatin content and distribution, and the location of nucleolus organizer regions (NORs) were analyzed. A comparative analysis of cytogenetic results also provided information on the possible karyotype evolution within the genus. Furthermore, we analyzed the presence of differentiated sex chromosomes, since it has been suggested, in the case of other species in the genus *Pseudacteon*, that sex determination is related to host size, and thus could be environmentally determined (Morrison *et al.*, 1999).

## Material and Methods

###  Fly rearing

Experiments were carried out between August, 2006 and November, 2007. *Pseudacteon* species were reared in colonies of *S. invicta* ants collected near Mercedes, Corrientes province (29° 47' S, 58° 03' W). Adult males and females of *P. curvatus* were captured in the Reserva Ecológica Costanera Sur (RECS), Buenos Aires province (34° 37' S, 58° 22' W), those of *P. tricuspis* near San Javier, Santa Fe province (30° 58' S, 59° 94' W), and those of *P. nocens* from Mercedes, Corrientes province (29° 11' S, 58° 5' W), all in Argentina.

###  Slide preparation

Different oviposition assays were performed in the lab following the same methodology used by Chirino *et al.* (2009). The “attacked” ants were placed in a rearing room at 28 ± 1 °C with a 12:12 (light: darkness) photoperiod and 80 ± 10% RH.

In Diptera, cytogenetic analyses are mainly carried out with third instar larvae or early pupae (Traut and Willhoeft, 1990; Cevallos and Nation, 2004). Since development in *Pseudacteon* takes place within its host (Porter *et al.*, 1995), it is difficult to obtain larvae at the appropriate developmental stages for cytogenetic studies. Thus we dissected pupae of 3-5 days, which lacked differentiated tissues, but showed high mitotic indices.

Since the females of *P. tricuspis* and *P. nocens* emerge from larger ant-heads than males, parasitized worker heads were measured and developed pupae were classified as either potential females or males. In contrast, both sexes of *P. curvatus* develop in similar sized hosts, so they could not be classified by sex (Morrison *et al.*, 1997; Folgarait *et al.*, 2006).

*Pseudacteon* pupae were dissected in a saline solution, swollen in a hypotonic solution (0.075 M KCl) for 10-20 min, and fixed for 15-30 min in freshly prepared Carnoy fixative (ethanol: chloroform: acetic acid, 6:3:1). Slides were prepared according to Traut (1976).

###  Chromosome morphology

Suitable cells at the mitotic prometaphase were selected and the mean descriptive values of the karyotype were calculated using information obtained from at least 3 cells per slide for each individual of each species analyzed (20.78 ± 9.76 cells). Each slide represented different individual pupae. Mitotic prometaphases were obtained from *P. tricuspis* pupae (17 males and 24 females); from *P. nocens* pupae (27 males and 21 females), and 35 specimens of *P. curvatus*. Slides were stained with the fluorescent dye DAPI (Rebagliati *et al.*, 2003) for morphological studies. The nomenclature of Levan *et al.* (1964) was used to describe chromosome morphology.

###  Banding techniques

Heterochromatin content and distribution was analyzed by means of the C-banding technique and sequential DAPI and CMA_3_ banding. C-bands were performed according to Sumner (1972) with slight modifications. Slides were hydrolyzed in HCl 0.2N at room temperature for 15 min, briefly washed in distilled water and then incubated at room temperature for 35 min in a 5% Ba(OH)_2_ solution. Slides were thoroughly washed in tap water for 1 min and incubated for 1 h in 2XSSC at 60 °C. After washing in distilled water, the slides were stained with DAPI (Rebagliati *et al.*, 2003), since band resolution improves when pre-treated slides are stained with DAPI instead of Giemsa. Fluorescent staining with GC specific chromomycin A_3_ (CMA_3_) and AT specific 4'6- diamidino-2- phenylindole (DAPI) was carried out according to Rebagliati *et al.* (2003).

For location of NORs, Ag-NOR staining was applied according to Howell and Black (1980). Fluorescent *in situ* hybridization (FISH) with 18S rDNA was also undertaken.

###  Fluorescence *in situ* hybridization

Unlabelled 18S rDNA probes were generated by PCR, using primers described by Fuková *et al.* (2005) and sequenced using an Automated Sequencer ABI 3100 (DNA Sequencer, PE, Applied Biosystems), thereby confirming their being a section of 18S rDNA of *Ceratitis capitata* (Weidemann) (Diptera: Tephritidae). PCR was done in a Mastercycler^®^ Gradient Eppendorf thermal cycler (Eppendorf AG, Hamburg, Germany). Reactions were carried out with template genomic DNA extracted from *C. capitata* by standard procedure according to Baruffi *et al.* (1995). The PCR product showed a single band of about 900 bp on a 1% agarose gel. The band was recovered from the gel and purified by using Wizard SV gel and PCR – clean up (Promega). The rDNA probe was labeled by nick translation with biotin 14-dUTP (BioNick Labeling System, Invitrogen Life Technologies Inc., Buenos Aires , Argentina). FISH with a biotinylated probe was carried out as described by Sahara *et al.* (1999) with several modifications as described by Fuková *et al.* (2005). Hybridization signals were detected with streptavidin-Cy3 conjugate (Sigma, Saint Louis, USA). The preparations were counterstained with 0.5 μg/mL DAPI in PBS, 1% Triton X-100, and mounted in anti-fade (Vectashield Mounting Medium, Vector Laboratories, Inc., Burlingame, USA).

###  Photographs

Preparations were observed in a Leica DMLB epifluorescence microscope. Black-and-white images of chromosomes were recorded with a CCD camera (Leica DFC350 FX, Leica IM50 Version 4.0, Leica Microsystems Imaging Solutions Ltd. Cambridge, UK) separately for each fluorescent dye. Images were pseudocolored (light blue for DAPI and red for Cy3), and, when necessary, superimposed with the aid of AdobePhotoshop version 6.0.

###  Statistical analysis

The following chromosome measurements were performed with MicroMeasure for Windows, version 3.3: centromeric index CI (length of short arm as a percentage of the whole chromosome), arm ratio r (relationship between the long and the short arms of each chromosome), relative length % (length of a chromosome as a percentage of the total chromosome length), and total chromosome length – TCL (the sum of the lengths of all the chromosomes of the complement). Comparisons of data within and between species were undertaken by analyses of variance (ANOVA), adjusted by the Bonferroni method. Statistical comparisons were done using the STATISTIX Program (Analytic Software 1998, Tallahassee, FL).

## Results

**Figure 1:**
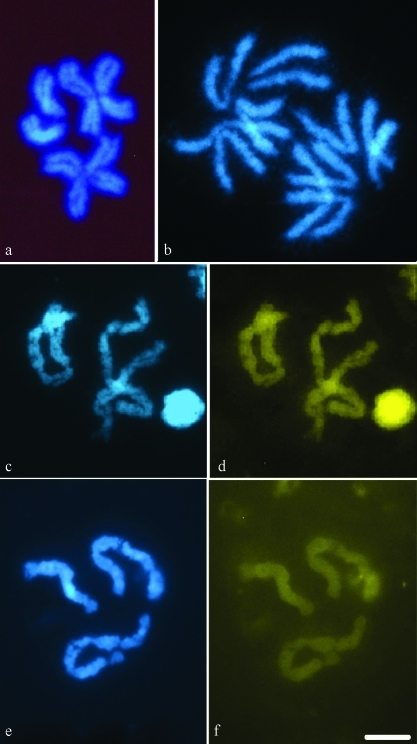
Cells in the mitotic metaphase (a) and anaphase (b) of *P. curvatus* stained with DAPI. Fluorescent banding with DAPI (c,e) and CMA_3_ (d,f) in *P. curvatus* (c-d) and *P. tricuspis* (e-f). Bar = 10 μm.

Analysis of mitotic chromosomes of the pupae of *Pseudacteon tricuspis, P. curvatus* and *P. nocens* revealed a 2n = 6 diploid number (Figure 1 a-b). Chromosome pairs I and II were metacentric in the three species, but chromosome pair III was subtelocentric in *P. tricuspis* and *P. curvatus*, and telocentric in *P. nocens* (Table 1). Neither *P. tricuspis* nor *P. nocens* possessed a distinguishable heteromorphic pair in potential males or females (*t* = 0.43, *df* = 39, p = 0.67 for *P. tricuspis* and *t* = 0.07, *df* = 46, p = 0.95 for *P. nocens*, 2-tailed *t*-Test). The three species did differ in total chromosome length (TCL) (*F*_2,99_ = 2.38, p = 0.0453) which was higher in *P. curvatus*, lower in *P. nocens* and intermediate in *P. tricuspis* (Table 2). Chromosome I in *P. nocens* was relatively larger than in *P. tricuspis* and *P. curvatus* (*F*_2,123_ = 8.68, p = 0.0004). Chromosome II in *P. nocens* was also larger than in *P. curvatus* (*F*_2,123_ = 5.86, p = 0.0039), whereas in *P*. *tricuspis* it was of intermediate length. Finally, chromosome III was smaller in *P. nocens* than in *P. tricuspis* and *P. curvatus* (*F*_2,123_ = 29.14, p < 0.0001) (Table 2).

**Figure 2:**
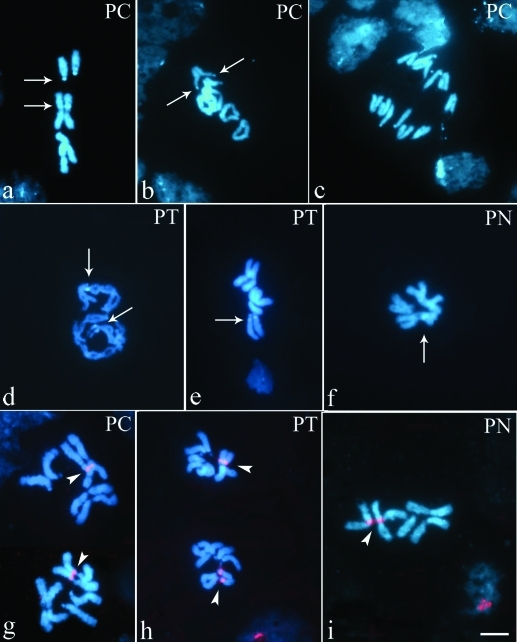
Somatic mitosis of *Pseudacteon curvatus* (a-c, g), *P. tricuspis* (d-e, h) and *P. nocens* (f,i) after C banding (a-f) and *in situ* hybridization with a 18S rDNA probe (g-i). At the top right-hand corner the names of species are indicated as PC = *P. curvatus*, PT = *P. tricuspis*, and PN = *P. nocens*. Arrows (a-f) indicate blocks of heterochromatin and head arrows (g-i), rDNA sites. Bar = 10 μm.

Different banding techniques were applied in order to reveal heterochromatin and analyze its location and composition. DAPI and CMA_3_ sequential fluorescent banding did not reveal any regions either in AT rich (DAPI bright) or GC rich (CMA_3_ bright) for any chromosome pair in any of the three species (Figure 1 c-f). After C-banding treatment, slides were stained with DAPI, allowing a better analysis of the C-heterochromatin pattern (Figure 2). C positive heterochromatin was scarce in all species, with a small C positive (C+) band in the long arm (q) of chromosome II and a completely C negative (C-) chromosome I (Figure 2a-f). “Somatic pairing” was evident from prophase up to metaphase (Figure 2a, d-i), but was lost during anaphase (Figure 2b-c). The three species varied as to the C-banding pattern in chromosome III. Thus, in *P. curvatus* a C+ band present in the short arm (p) extended over 81% of its length (Figure 2a-c). In *P. tricuspis*, a C+ band observed in the long (q) arm near the centromere corresponded to approximately 4% of its length (Figure 2d-e). Finally, the telocentric pair in *P. nocens* was completely C- (Figure 2f).

**Figure 3:**
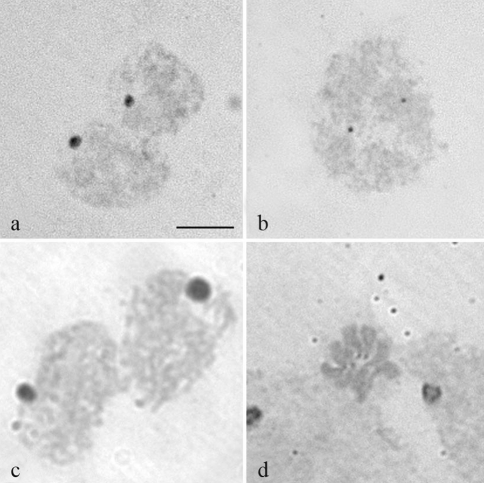
Interphase nuclei of *P. curvatus* (a) and *P. tricuspis* (b), and the early prophase (c) in which one nucleolus was observed after silver nitrate NOR banding. d) Metaphase of *P. tricuspis*. No Ag-NOR band was detected on the condensed chromosomes. Bar = 10 μm.

**Figure 4:**
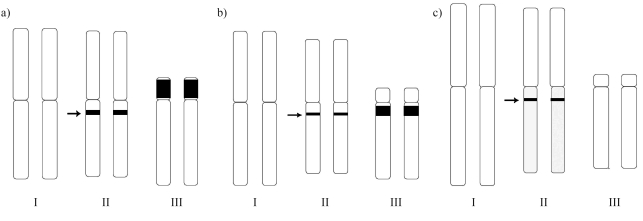
Idiograms of *Pseudacteon curvatus* (a), *P. tricuspis* (b) and *P. nocens* (c). C-bands are shown as black segments, and arrows point out the location of the hybridization signal corresponding to18S rDNA.

Silver impregnation revealed only one nucleolus in interphase nuclei and two signals in early prophase cells in the three species. However, no positive results were obtained in metaphase chromosomes (Figure 3). *In situ* hybridization with the 18S rDNA probe on metaphase cells showed a single hybridization signal in chromosome II in the three species at a location similar to that of the C+ band (Figure 2g-i; Figure 4). This suggests that in the three *Pseudacteon* species a single cluster of rRNA genes is located in the long arm (q) of chromosome II near the centromere.

## Discussion

The *Pseudacteon* species analyzed presented 2n = 6, with chromosomes I and II being metacentric and chromosome III subtelocentric in *P. curvatus* and *P. tricuspis* but telocentric in *P. nocens.* Total chromosome length (TCL) varied among *Pseudacteon* species, with *P. curvatus* ≥ *P. tricuspis* ≥ *P. nocens*, probably representing variations in DNA content that do not affect chromosome morphology. A similar situation has been reported in other dipteran families (Rafael and Tadei, 1998; Parise-Maltempi and Avancini, 2001; Selivon *et al.*, 2005).

**Figure 5:**
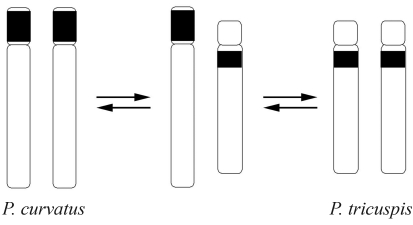
Hypothesized karyotype evolution in *Pseudacteon curvatus* and *P. tricuspis*. Differences in chromosome III can be explained by pericentric inversion, although it is not possible to determine the direction of evolutionary change.

Heterochromatin content was very scarce in these *Pseudacteon* species. Chromosome I was completely C-, with a single C+ band situated at the location of the 18S rDNA hybridization signal on chromosome II. Major differences were observed in chromosome III, with a C+ band in the p-arm of the subtelocentric pair in *P. curvatus* and the q-arm in *P. tricuspis*, although this was absent in the telocentric pair of *P. nocens* (Figure 4). The divergence between the former two species could be explained by pericentric inversion in chromosome III during their evolutionary history (Figure 5). Its absence in *P. nocens* suggests that it could have diverged before the differentiation of *P. tricuspis* and *P. curvatus*. Furthermore, the smaller size of chromosome III in *P. nocens*, as compared to the other two species, could be explained by the absence of a heterochromatin block. Although it would be necessary to analyze other *Pseudacteon* species, we propose here a possible hypothesis on karyotype evolution within the genus. We propose that after the divergence of *P. nocens*, and through the acquisition of heterochromatin, there was an increase in DNA across the genome of the ancestor of the other two species, but principally in chromosome III. In spite of the fragmented and unresolved phylogenies of some *Pseudacteon* species (Kronforst *et al.*, 2007; Calcaterra *et al.*, 2008), several conclusions support our hypothesis. The recent common ancestor of *P. nocens* is different from the most recent common ancestor of *P. curvatus* and *P. tricuspis* (Calcaterra *et al.*, 2008), and phylogenies based on the mitochondrial genes *Cytochrome Oxidase I* and *II* and the nuclear gene *Wingless* (Kronforst *et al.*, 2007) suggest that *P. tricuspis* could have derived from the ancestor from which *P. curvatus* diverged.

In reference to the studied *Pseudacteon* species, Ag-NOR banding revealed the presence of one nucleolus but not on metaphase chromosomes. Similar observations have been reported in other dipterans in which results from silver staining were unsatisfactory, and only centromeric cores could be differentiated (Motara *et al.*, 1985; Wallace and Newton, 1987; Bedo and Webb, 1989; Marchi and Pili, 1994).

Cytogenetic characterization of *Pseudacteon* species failed to reveal morphologically differentiated sex chromosomes. The only other two species in the Phoridae in which cytogenetical data has come under analysis were *Megaselia scalaris* and *M. spiracularis.* Accordingly, both possess the same diploid number 2n = 6 and a similar chromosome morphology, with two metacentric and one telocentric chromosomes (Mainx, 1964). In this genus sex determination depends on the presence of a single *Maleness* factor M that triggers off male development (Mainx, 1964; Willhoeft and Traut, 1990; Traut and Wollert, 1998; Traut *et al.*, 1999). Through molecular cytogenetic techniques and molecular markers it has been established that the chromosomes X and Y in *Megaselia* are in a very early state of molecular differentiation (Traut, 1994).

In most of the studied *Pseudacteon* species, the sex of the offspring seems to be facultatively determined by the size of the host, with females emerging from the larger ones (Morrison *et al.*, 1999; Folgarait *et al.*, 2005a, 2006). From an evolutionary point of view, this makes sense since 1) fire-ant workers are highly variable in size (Tschinkel *et al.*, 2003), 2) female flies have a higher fitness if they emerge from larger hosts, exhibiting a higher efficiency in development and larger sizes (Chirino, Gilbert, Folgarait, unpublished), and 3) females emerge from a narrower range of ant-sizes than males (Folgarait *et al.*, 2005a, 2006; Chirino, Gilbert, Folgarait, unpublished). However, in two small species, *P. cultellatus* Borgmeier (Folgarait *et al.*, 2002) and *P. curvatus* (Chirino *et al.*, 2009), females and males develop in similar-sized hosts, and emerged adults are not sexually dimorphic in size, so that the above mentioned pattern is difficult to detect. The number of different species of *Pseudacteon* that develop on *S. invicta* exceeds the diversity observed in other ants (Disney, 1994). This diversity could be influenced by *Solenopsis* size polymorphism since there is a correlation between the sizes of both parasitoid and host (Morrison *et al.*, 1997). Different *Pseudacteon* species oviposit at different times during the day, at different locations (close to mounds or the foraging trails of ants) and during different seasons. *P. nocens* in particular is crepuscular and abundant in shady conditions, but *P. curvatus* and *P. tricuspis* are more abundant at midday and in sunny conditions (Folgarait *et al.*, 2007a; 2007b). Furthermore, *P. curvatus* mainly attacks small workers (Chirino *et al.*, 2009), *P. tricuspis* larger ants (Morrison *et al.*, 1997), and *P. nocens*, medium-sized ones. Finally, *P. tricuspis* females exhibit competitive, aggressive and territorial behaviors, which are not observed in either *P. curvatus* or *P. nocens*. In this work we have shown that the well-known differences among *P. curvatus, P. nocens* and *P. tricuspis* as regards size, morphology, behavior and phylogenetic relations are also reflected in their karyotypes.

The exact mechanism of sex determination in *Pseudacteon* flies is perplexing (Porter, 1998). Environmental sex determination (ESD) has been suggested as a possible mechanism for the observed pattern (Morrison *et al.*, 1999). Typically, taxa having non-differentiated sex chromosomes usually show labile sex-determination, and sex can be determined by environmental stimuli (Solari, 1994). Our demonstration of non-differentiated sex chromosomes in *Pseudacteon* phorids is thus consistent with the earlier suggestion of environmental sex determination in these parasitoid flies, and should encourage further investigation of how sex is determined in this genus.

## Figures and Tables

**Table 1:** Comparison of the somatic complement of the *Pseudacteon* species analyzed. %: relative length, r: arm ratio, CI: centromeric index, N: number of slides analyzed.

*Pseudacteon curvatus*						
Chromosomes	Average length (μm)^1^	%^1^	r	CI	Classification	N
I	47.11 ± 17.53a	19.96a	1.14 ± 0.10	46.87	m	
II	42.19 ± 14.89a	17.75b	1.15 ± 0.09	46.63	m	35
III	30.01 ± 12.14b	12.29c	3.99 ± 0.89	21.18	st	

*Pseudacteon tricuspis*						

Chromosomes	Average length (μm)	%	r	CI	Classification	N

I	44.69 ± 11.14a	19.88a	1.20 ± 0.17	45.78	m	
II	40.43 ± 9.26a	18.07b	1.18 ± 0.16	46.20	m	41
III	27.20 ± 7.77b	12.05c	5.20 ± 0.74	14.53	st	

*Pseudacteon nocens*						

Chromosomes	Average length (μm)	%	r	CI	Classification	N

I	41.21 ± 10.12a	20.76a	1.14 ± 0.08	46.94	m	
II	36.64 ± 8.88b	18.48b	1.13 ± 0.07	46.98	m	48
III	21.53 ± 5.56c	10.75c	4.91 ± 0.60	12.20	t	

^1^The comparisons of chromosomal lengths and the relative lengths (%) were made by means of one-way ANOVA. Different letters indicate significant differences (p < 0.05) adjusted by Bonferroni.

**Table 2:** Comparisons of total chromosomal lengths (TCL) and relative lengths (%) among the *Pseudacteon* species analyzed.

Species	TCL (μm) ^1^	%		
		I	II	III
*P. curvatus*	225.31 ± 70.74 A	19.96 B	17.75 B	12.29 A
*P. tricuspis*	220.11 ± 54.69 AB	19.88 B	18.07 AB	12.05 A
*P. nocens*	198.45 ± 46.01 B	20.76 A	18.48 A	10.75 B

^1^The comparisons of the total chromosomal lengths (TCL) and the relative lengths (%) were analyzed by means of one-way ANOVA. Different letters indicate significant differences (p < 0.05) adjusted by Bonferroni.
